# Tracking Rural Health Facility Financial Data in Resource-Limited Settings: A Case Study from Rwanda

**DOI:** 10.1371/journal.pmed.1001763

**Published:** 2014-12-02

**Authors:** Chunling Lu, Sandy Tsai, John Ruhumuriza, Grace Umugiraneza, Solange Kandamutsa, Phillip P. Salvatore, Zibiao Zhang, Agnes Binagwaho, Fidele Ngabo

**Affiliations:** 1 Brigham and Women's Hospital, Harvard Medical School, Boston, Massachusetts, United States of America; 2 Brigham and Women's Hospital, Boston, Massachusetts, United States of America; 3 Partners In Health/Inshuti Mu Buzima, Rwinkwavu, Rwanda; 4 Ministry of Health, Kigali, Rwanda

## Abstract

Chunling Lu and colleagues describe a project for tracking health center financial data in two rural districts of Rwanda, which could be adapted for other low- or middle-income countries.

*Please see later in the article for the Editors' Summary*

**Summary Points**

- Tracking financial data for rural health facilities is difficult in low-income countries because of unstandardized accounting practices and the absence of effective health financial information tracking systems.
- Poor-quality financial data hinders monitoring and evaluation of health facility performance.
- We present a five-step procedure developed for gathering financial data from 21 health centers in two rural districts of Rwanda.
- The five-step procedure generated financial data with internal consistency and a low percentage of reports of “missing” for in-kind support (donated goods and services). In-kind support (mainly medicine and equipment) accounted for a large proportion of the total expenditure of health centers.
- We report challenges faced by the project and make suggestions for how Rwanda's national web-based financial data collection system can be improved.
- Knowledge gained from the Rwanda field experience may inform other low-income countries on how to establish an information system to track health facility financial data.

## Background

Rural health facilities in low-income countries play key roles in providing accessibility to quality care to the majority of their populations. Timely, reliable, and comparable financial data from rural health facilities is critical for making effective financial projections, ensuring sufficient and sustainable funds, and monitoring and evaluating the performance of health facilities [1],[2]. Tracking financial records in low-income countries is known to be difficult because of poor accounting practices and a lack of standardized internal auditing and financial reporting [3]. The difficulty is amplified by the absence of effective health information systems (here defined as health facility financial data tracking systems) in rural areas, resulting in irregular or incomplete financial records. As was noted by the World Health Organization (WHO) in 2010, “few developing countries have sufficiently strong and effective health information systems to meet their information needs” [4].

Rwanda, a country with a gross domestic product per capita of US$595 in 2011 [5], has 448 health centers that serve about 85% of its population, who live in rural areas [6]. To improve health service delivery in rural areas, Rwanda adopted a decentralized health financing policy in 2006 and granted managerial autonomy to health facilities in administrative districts. The shift of fiscal and managerial responsibilities from the central Ministry of Health (MoH) to local health facilities created a high demand for quality data at the facility level for financial planning and performance evaluation [7]. One of the major challenges for implementing decentralization was the lack of evidence-based performance evaluation for health facilities [8]. To address the issue, the MoH built a web-based database system, the District Health System Strengthening Tool (DHSST), which requires hospitals and health centers in the public sector to report various indicators, such as service delivery and finances, to the database on an annual basis [9]. The DHSST posts an online standardized survey tool, including a section about health facility finances and expenditures, which allows accountants in each health facility to log into the online survey and report the facility's annual spending and the funds it received. While the online reporting system is a useful channel for gathering financial data, it was found to be ineffective in capturing the value of in-kind support (donated goods and services) received by the health centers. We found that, among the 438 health centers that reported to the DHSST in 2011, 357 (82%) reported “missing” for received medicine and consumables. One aim of the initiative reported in this article is to effectively improve online financial data collection and to reduce the missing in-kind support information reported by rural health centers.

We investigated the methods that have been previously used for collecting financial data in local health facilities in resource-poor settings and searched for and reviewed relevant studies published in peer-reviewed journals from 2000 to 2012 [10]–[19]. While many studies focused on costs or cost-effectiveness analyses of a specific intervention, few of them described how the cost data were generated. Values of in-kind support were often neglected because of unavailable information. Little research has been conducted on developing effective health information systems for tracking financial data at local health facilities in low-income countries.

As part of an economic evaluation of the Rwanda Population Health Implementation and Training (PHIT) Partnership, this article describes a project of tracking health center financial data in two rural districts of Rwanda: Kirehe, and the southern area of Kayonza. The PHIT Partnership is a five-year project that was established in 2009 to implement a comprehensive district-level health systems strengthening model in the two rural districts [20]. Southern Kayonza and Kirehe are contiguous over an area of roughly 3,000 km^2^ in southeastern Rwanda, with a population of 480,000 people. Health services are delivered by two district hospitals at the district level and 21 health centers at the sector level. The district hospitals provide secondary care with services such as inpatient care, minor and major surgery, laboratory analyses, and medical imaging. The health centers deliver primary care for promotional activities (campaigns on child growth monitoring, knowledge of nutrition and hygiene, etc.), preventive activities (vaccination, prenatal and postnatal care, family planning, etc.), and curative activities (nutritional rehabilitation, integrated management of childhood illness, normal deliveries, HIV treatment, etc.).

## Five-Step Data Tracking Procedure and Its Implementation

We report a five-step financial data tracking procedure that was conceptualized at the pilot stage and fully developed during the process of collecting financial data from the 21 health centers in the two districts. WHO's guidance on producing national health accounts [3] helped us design the procedure to deal with the challenges of financial data tracking in resource-limited settings. The collected financial data include health centers' annual expenditures as well as their annual funds received from various sources. The procedure for tracking financial data and how it was implemented is described below.

### Step 1: Understanding Channels of Resource Flows of Health Centers

**Figure 1:**
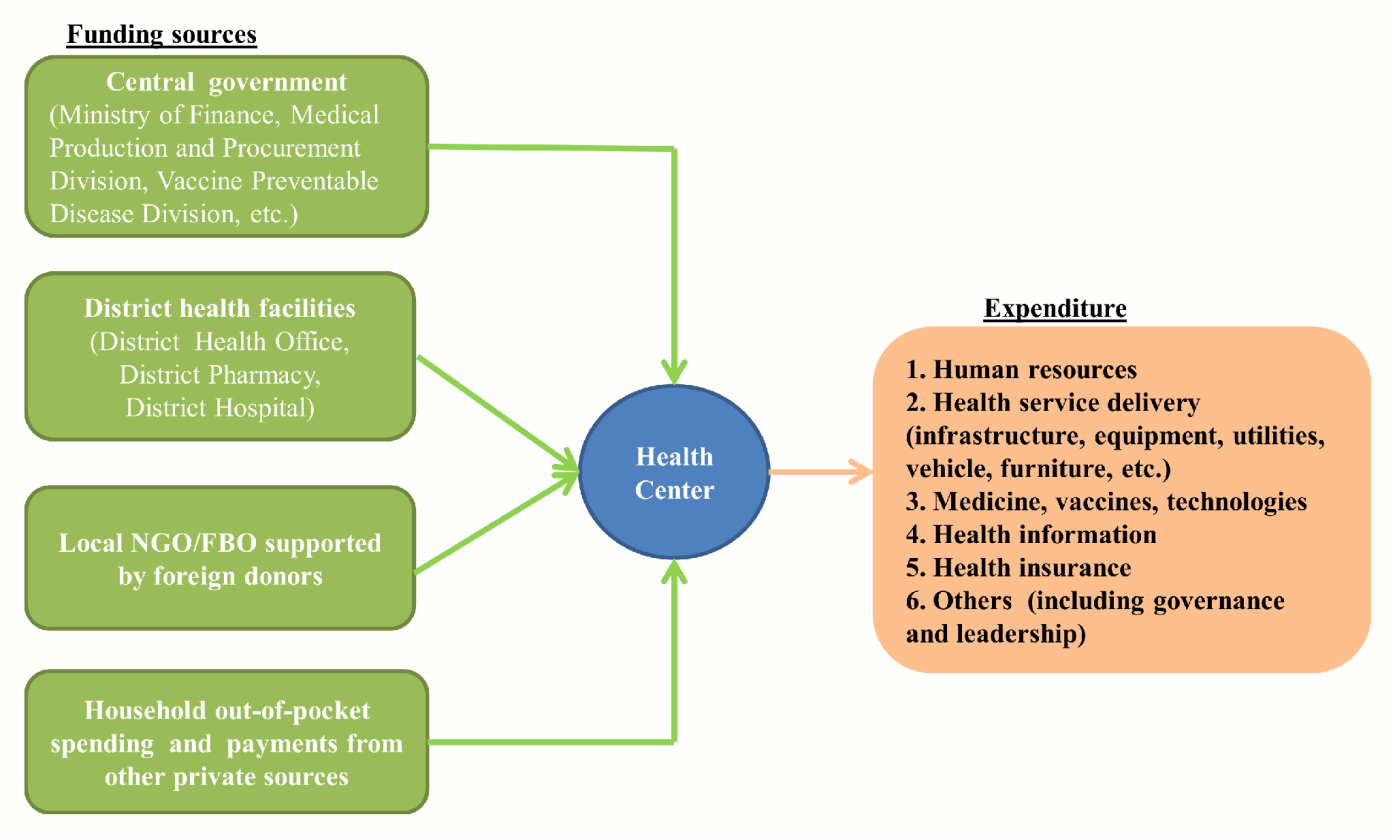
Channels of resource flows for health centers in Kirehe and the Southern Kayonza.

Rwanda has been undergoing rapid health system reform since 2006. To gather reliable and comparable health financial data from the health centers in the two districts, it is important to obtain the most up-to-date information about the health system structure in the area and to understand the interactions between health centers and other health system actors. We reviewed all available policy documents on health system reform and its effect on the structure of the health sector at the national and district levels. We paid special attention to the mechanisms of health sector financing. The documents were provided by the MoH and the two district health offices. We interviewed health management officials and accountants at the district and health center levels to identify other health system actors in their respective areas and the channels of resource flows to health centers. We found that, in addition to the 21 health centers and the two district hospitals, there are two district health offices, two district pharmacies, and various health-focused nongovernmental organizations (NGOs) and faith-based organizations (FBOs) supported by foreign donors. Health centers in the two districts received cash or in-kind support from three major sources: (1) public health agencies (at the central and district levels), (2) external donors (including donor-supported NGOs and FBOs), and (3) private health spending, primarily households' out-of-pocket health payments (Figure 1).

### Step 2: Identifying Financial Data Sources

Information derived from Step 1 helped us in identifying financial data sources. By interviewing health management officials and accountants at the central and local levels, we found that, while cash flow information could mostly be obtained from health centers, much of the in-kind support data had to be gathered from their providers (Box 1). For example, in 2009 and 2010, health centers received most of their medicine (without making payments) directly from the Rwandan Medical Production and Procurement Division (MPPD), Rwandan Vaccine Preventable Disease Division (VPDD), and the district pharmacies. Health centers had no records about the transactions, and the providers kept the delivery notices. By relying solely on the reports from health center accountants (as the DHSST does), the value of unpaid-for medicine will not be captured and, as a result, drastically underestimated.

**Box 1. Data Sources for Cash and In-Kind Support Received by the 21 Health Centers**

**Cash**

1. Health center's financial records and its health management information system monthly reports
2. Records from foreign donors (such as Partners In Health, the Global Fund to Fight AIDS, Tuberculosis, and Malaria, etc.) when they are available

**In-kind support**

1. Transaction records from central or district public health agencies, such as the Medical Production and Procurement Division, the Vaccine Preventable Disease Division, and district pharmacies.
2. Transaction records from foreign donors (such as Partners In Health and the Global Fund to Fight AIDS, Tuberculosis, and Malaria) when they are available

### Step 3: Designing Survey Instruments

To collect expenditure data for health centers, we designed survey instruments with a bottom-up approach recommended by WHO [3]: obtaining spending information on individual elements and aggregating them into the total. Our expenditure survey was structured around the WHO framework [21] that describes a health system in terms of six core components or “building blocks”: (1) health service delivery, (2) health workforce, (3) health information systems, (4) essential medicines, vaccines, and technology, (5) financing, and (6) leadership and governance. Under each category, a list of questions was asked regarding cash and in-kind expenditures. A breakdown of reported costs into these six building blocks makes it possible to assess whether or not the resources are efficiently allocated among the six building blocks for delivering quality services.

When designing questions to capture in-kind support, we adopted the “ingredients approach” [22],[23] and asked health centers to report the donated item's name, provider's name, quantity, unit price, and percentage of usage, rather than the total value of in-kind donations as the DHSST does. The differences between the two approaches are summarized in Table 1. The “ingredients approach” allowed us to obtain more information to identify more data from health centers. For example, health centers usually did not have regular records for received unpaid-for medicine. Based on the names of providers, we were able to track down the data from the providers (MPPD, VPDD, etc.). If health centers only reported the quantity of an item, we obtained its value using protocols developed for cost estimation (Text S2).

**Table 1:** Summary of different approaches in reporting in-kind support by the DHHST and the five-step procedure: an example.

**District Health System Strengthening Tool Survey**				
*Donated items*	*Value of donations*	–	–	–
Office furniture	__(Rwanda Francs)	–	–	–
	__Do not know.			
**Five-Step Procedure**				
*Donated items*	*Donor's name*	*Quantity*	*Unit Price*	*Percentage of usage*
Office furniture	__	__	__ (Rwanda Francs)	__ (%)
	__ Do not know.	__Do not know.	__Do not know.	__Do not know.

It is important to note that survey design is an iterative process, partly as a result of self-learning and partly as a result of rapid changes in financing structures in health centers. The process of survey development included the following: (1) designing and piloting the first draft of the questionnaire, (2) revising the questionnaire based on the pilot study and feedback from the accountants, (3) applying the revised questionnaire to all health centers, and (4) revising the questionnaire in response to any new changes reported by data collectors and respondents.

### Step 4: Building up Local Capacity for Data Collection

Building a local data team is crucial for collecting high-quality financial data. Our Rwanda data collection team was comprised of one research coordinator and two data collectors with college degrees from universities in Rwanda. We designed a series of training sessions for the team so that the team members could understand research objectives and survey instrument design and acquired skills in interviewing respondents, data tracking, data storage, data entry and cleaning, and quality control. The training sessions on each topic were given either before or during the first three months of field work, after which the team could independently implement data collection and management with minimal support from senior researchers.

To ensure the quality of data and to promote positive interactions between the data collectors and accountants, we obtained approval from the district health offices to conduct a two-day workshop to orient accountants before each cycle of data collection. We distributed the survey, which included a section for accountants to report their comments on survey questions, to accountants during the workshop. The trainings and consultations not only helped them in completing the survey but also gave them a better understanding of the importance of obtaining quality data and increased their sense of ownership over the project. After completing the data analysis, we disseminated the findings to each health center and encouraged them to give us their feedback on the survey and use the information for their financial planning. This process allows for an ongoing dialogue between data collectors and health center staff, establishes a mechanism for updating the survey if any changes occur, and promotes results-based resource allocation.

### Step 5: Implementing Data Collection

Based on the data sources identified in Step 2, we gathered data from multiple sources: health centers, district health facilities, and central health institutions. Information about cash flow was collected directly from the accountants of the health centers. The data collectors visited the accountants' offices to help them complete the survey. Together, they went through the facility's financial records and checked the quality of the reported data. Follow-up calls or visits were made if it was necessary. In the first year of the project, the average time for completing data collection in a health center was about 15 hours with an average of three to four visits. The time to collect the data is expected to be reduced in subsequent years as both the data collectors and accountants become more efficient in completing the survey.

With approval from the MoH and district-level health offices, information about unpaid-for medicine (including vaccines and contraceptives), consumables, and equipment was collected directly from the MPPD, VPDD, district pharmacies, and donors. Information on other in-kind support was obtained from the health centers or the local NGOs/FBOs if written records were available. When no written records were available for an item, a missing value was assigned to the item. When information collected from multiple sources did not agree, we discussed the issue with the data sources through phone calls and reconciled the records. For example, if a health center's records on received computers from Partners In Health (PIH) were different from the records provided by PIH, we called both parties to determine the cause of the difference and the appropriate values. To check the consistency of the reported data from a health center, we aggregated its reported expenditures and funds received in a fiscal year and compared the total values of these two items.

Most health centers were only able to provide quantity information of in-kind support. To obtain the values of these items, we developed an estimation protocol based on market prices or known costs of similar items reported by other health centers or health institutions.

Data entry and cleaning took place simultaneously with data collection on a weekly basis, allowing for quality control.

Survey instruments and cost estimation protocols are provided in the Supporting Information (Text S1, Text S2).

## Evaluation of the Five-Step Procedure

We evaluated the five-step data tracking procedure by examining (1) the proportion of in-kind data that was reported missing and (2) the consistency between the aggregated received funds and aggregated expenditures reported by the health centers.

### (1) Missing Data of In-Kind Support

Both the DHHST and five-step procedure surveys asked the health centers to report received in-kind support. The DHHST listed six items in its survey, and the five-step procedure listed 14 items in the survey. Table 2 presents the missing proportions of four groups of items that were both included in the surveys of the DHHST and the five-step procedure. Items that only appeared in one of the two surveys were not included. We calculated the percentage of the 21 health centers that reported missing information for the items in these four groups. For the 21 health centers, the DHSST data in fiscal year 2010 demonstrated much higher rates of missing values in the four listed items in the Table 2, from 52% for donated vehicles to 91% for donated medicine. In comparison, missing rates in the data generated from the five-step procedure ranged from 7% for donated medicines to 17% for donated vehicles.

**Table 2:** Missing proportions of in-kind donations in the 21 health centers.

Fiscal Year	Category	DHSST	Five-Step Procedure
2010	Medicine (including vaccines and contraceptives)	91%	7%
2010	Equipment (medical and nonmedical)	57%	15%
2010	Office furniture	57%	13%
2010	Vehicles	52%	17%

In-kind donations accounted for a large proportion of the total expenditures of health centers. For example, in fiscal year 2010, the average percentage of in-kind support in total expenditures was about 46%, with a range from 31%–72% across the 21 health centers (Figure S1). Donated medicine and equipment made up 75% and 84% of all in-kind support in the 21 health centers in fiscal years 2009 and 2010, respectively. Donations of infrastructure (such as buildings, renovations, furniture, vehicles, information technology, utilities, and office equipment) accounted for the second largest proportion (22% in 2009 and 10% in 2010) of in-kind support. Donations to human resources and others were 3% in 2009 and 7% in 2010 (Figure S2).

### (2) Internal Consistency

We validated the internal consistency of the data by comparing a health center's reported total received funds with its total expenditures. If the ratio of total expenditures to total funds received approximates to 1, the data can be considered balanced and as reaching internal consistency. The ratio ranged from 0.74 to 1.22 in fiscal years 2009 and 2010 (Figure S3). More than 70% of health centers had ratios between 0.9 and 1.1 in the two years, suggesting a close match of the two aggregated estimates.

## Challenges and Recommendations

We encountered several challenges when implementing the five-step procedure. The 21 health centers did not have standardized tools for recording cash and in-kind support, which made data collection burdensome to both the accountants and data collectors. Data reporting was not a part of the accountants' routine work, and the data submission was frequently postponed or left blank by the accountants. To ensure the completion of the survey, data collectors had to make multiple follow-up phone calls or visits.

Accurate and reliable financial data is essential for efficiently allocating resources across the six building blocks of a health system. The Rwandan MoH considers an effective health information system to be a critical backbone of its strategic planning and has committed to strengthening its health information systems by establishing online data collection tools at the district level in recent years. The costing project of the PHIT Partnership is limited in terms of the time span (five years) and scope (two rural districts). However, it has developed and validated a financial data tracking procedure that can be applied in other rural districts of Rwanda. The evidence generated from our field experience contributed to the strengthening of health information systems in Rwanda by serving as an independent assessment of the DHSST in tracking financial data at the national level. More importantly, we recommend that the five-step procedure be integrated into the Rwandan national health information system to augment its capacity for tracking financial data at the district level. We anticipate several key advances and improvements to come from this project and are working closely with the Rwandan MoH to incorporate our findings.

The most immediate action that the MoH plans to take is to improve the existing online system of tracking financial data by informing the DHSST team of the limitations of its financial data collection tool and urging the DHSST to adopt the ingredients approach for tracking in-kind donation. In order to increase the response rate for in-kind support, we recommend that the DHSST adopt the ingredients approach and expand data collection from health centers to other related sources (such as the MPPD and VPDD).

To further improve data quality and reduce the costs and time needed for data collection, the short-term priority for the MoH is to strengthen the facility-level information reporting system by developing a standardized and easy-to-use financial recording book to document both cash and in-kind transactions in health centers. The survey instruments of the five-step procedure will be used as a reference in designing the new financial recording book for use by all accountants at the health facilities. With the support of the Ministry of Finance, the MoH will coordinate with health centers, the DHSST team, and other stakeholders to adopt the new recording system and make data reporting a regular duty of health center accountants.

In the long term, the government of Rwanda has committed to devote at least 15% of total government spending to the health sector, based upon the fact that the GDP growth rate in Rwanda has been between 6% and 8% in the past decade. With the support of the Ministry of Finance and Economic Planning, the MoH will allocate appropriate resources and staffing at both the central and facility levels through decentralization. This will provide sustainable support to integrate the five-step procedure into the existing national-level health information system. In addition to ensuring there are adequate computers, information technology (IT) equipment, and office infrastructure, the process will engage both data producers and users in obtaining the necessary knowledge and skills in tracking and using quality financial data. Data producers will be trained to develop their capacity for data collection, management, assessment, analysis, and dissemination. Health facilities will be required to make more efficient use of the data for planning based on the evaluation of the impact of their expenditures on population health outcomes. Strategic planning of these long-term activities will be a component of the Health Information System Program in Rwanda. Under the decentralization policy, districts will receive more funds from the central government in the long term, which will enable them to build capacity for financial data management. The time frame for scale-up is under discussion between the central MoH and local districts.

## Lessons Learnt

Our experience in these two rural districts of Rwanda suggests that by applying the presented five-step data collection procedure, the quality of financial data can be significantly improved, even in a context with very limited resources. This is encouraging for other low-income countries that are in similarly challenging situations.

Like Rwanda, many low-income countries have been undergoing health sector reform by decentralizing health financing and delegating decision-making to local health facilities [24]–[26]. Local health facilities need to have timely and reliable data about their finance and service delivery for effective budgeting, reporting, and planning. Meanwhile, health-focused development assistance to low-income countries (as cash or in-kind donations) has increased drastically over the past decade to support these countries in meeting the health-related Millennium Development Goals [27]. According to the Global Health Expenditure Database by WHO, health aid made up 20% to 56% of total health expenditures in 24 sub-Saharan countries in 2010 (47% in Rwanda) [28]. External health aid flows into these countries at both the central and local levels and has increased pressure on recipients and stakeholders for regularly documenting financial inputs and expenditures to ensure the efficient use of limited resources. Donors, such as the Global Alliance for Vaccines and Immunization (GAVI) and the Global Fund to Fight AIDS, Tuberculosis, and Malaria (GFATM), support sub-Saharan countries mostly with goods and services (bed nets, vaccines, medicine, equipment, training, etc.). Our calculation with data from the Organization for Economic Co-operation and Development [29] shows that in-kind support from GAVI and GFATM accounts for 37% of total health aid for low- and lower-middle-income countries in the sub-Saharan region, suggesting that the five-step procedure could be a useful tool for these countries in tracking both cash and in-kind expenditures at health facilities.

The lack of capacity to produce and use quality financial data for informed policy making is a challenge faced by many developing countries. In recent years, Rwanda has been making substantial efforts to build the country's health information system for results-based resource allocation and has taken a lead in adopting district-level health information systems among sub-Saharan countries. The country hosted international training sessions on strengthening information systems for health officers from 13 sub-Saharan countries and countries from other regions in May 2014 [30]. Our experience in developing and practicing the five-step procedure in these two rural districts provides a method for generating reliable and complete financial data in rural Rwanda. Lessons learnt could contribute to other low-income countries in establishing or strengthening their health information systems.

Our experience shows that strong government commitment to improving health information systems at all levels is necessary for the success of the five-step procedure. During the implementation process, we received support from the central MoH and the district health offices to obtain financial records from the central- or district-level health institutions. These records were the main sources of data for in-kind items. The timely responses of these institutions ensured the on-time completion of the project. Training accountants was found to be helpful in cultivating ownership of the project and improving data quality. However, having accountants leave for a two-day workshop could be inconvenient for health centers. We obtained permission from the district health offices and the directors of the health centers to do the training. We found that accountants who received support from their directors for financial data reporting were more likely to finish the survey on time with fewer errors.

The analysis of the collected data shows that in-kind donations accounted for a substantive proportion of health center expenditures. This indicates the importance of including in-kind support in reported facility data in countries that receive large quantities of items such as medicine and vaccines, bed nets, and medical equipment from international donors. As indicated by the Global Health Expenditure Database, in more than half of the sub-Saharan countries, health aid made up 20% or more of their total health expenditures in 2010 [28]. The absence of in-kind items in financial data may severely underestimate the expenditures of health facilities in those countries and lead to biased financial planning and performance evaluation.

Although the survey instruments and cost estimation protocols provided by this project are for rural health centers in Rwanda, the underlying framework and the majority of content can easily be adapted to other facilities (such as hospitals or pharmacies) or other relevant programs (curative care, preventive care, etc.) in other countries. The supplemental survey instruments were designed to answer important policy questions such as the following:

1. How much did the government contribute to health facility financing? The estimates could be used as an indicator for a government's commitment to health.
2. How much did the external aid contribute to a health facility's overall expenditure? The estimates could help us understand the sustainability of existing services.
3. Were the resources allocated efficiently across the six building blocks?
4. Did a high level of expenditure lead to an increase in medical service coverage in both quantity and quality and ultimately improve population health?

For policy makers, donors, or other stakeholders who are interested in these questions at the health facility level, the five-step procedure, its survey instruments, and its cost estimation protocols could serve as good references for building systems to gather quality finance information from health facilities.

In summary, quality financial data are essential for health policy design and implementation, as well as for monitoring and evaluation. Conditional upon a government's commitment to evidence-based decision making and with a clear understanding of local health systems, careful design of data collection, and investment in building local human capacity, a financial data tracking system can be established in resource-poor settings.

## Supporting Information

Figure S1 **Proportion of in-kind support in the total expenditure of the 21 health centers.**
(TIF)

Figure S2 **Composition of in-kind support in the 21 health centers.**
(TIF)

Figure S3 **Ratio of total expenditures to total funds received across the 21 health centers.**
(TIF)

Text S1 **Health center financial survey.**
(DOCX)

Text S2 **Cost estimation protocols.**
(DOCX)
