# Supplementary figures and images for: Tracking Rural Health Facility Financial Data in Resource-Limited Settings: A Case Study from Rwanda
Source: PLoS Med. 2014 Dec 2;11(12):e1001763. doi: 10.1371/journal.pmed.1001763 (PMC4251825; doi:10.1371/journal.pmed.1001763)

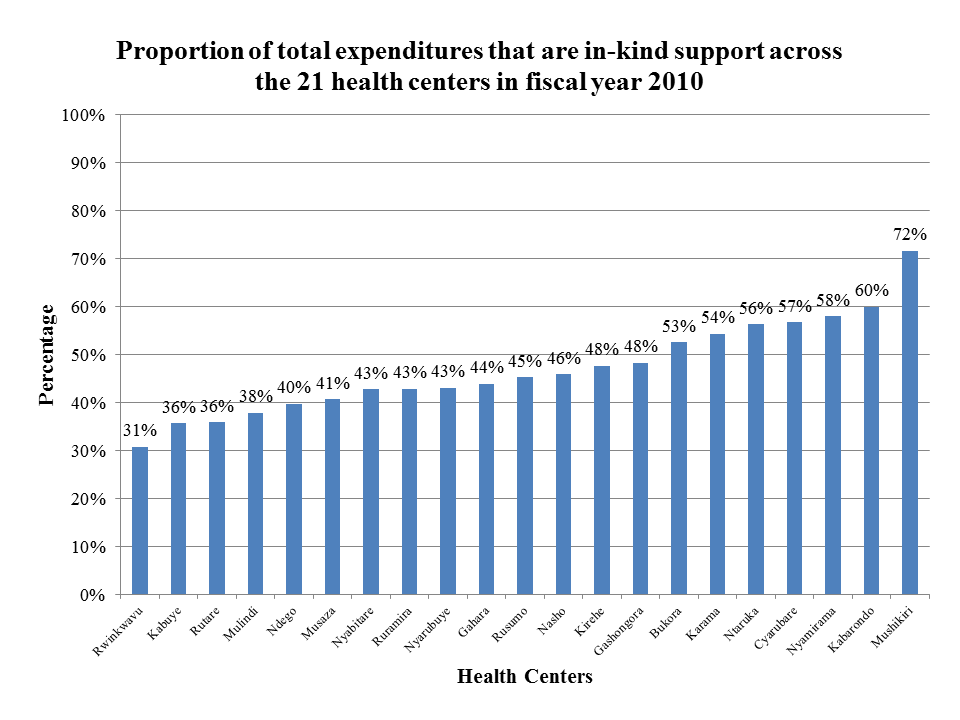

Supplement: Figure S1 — Proportion of in-kind support in the total expenditure of the 21 health centers. (TIF) [file pmed.1001763.s001.tif]

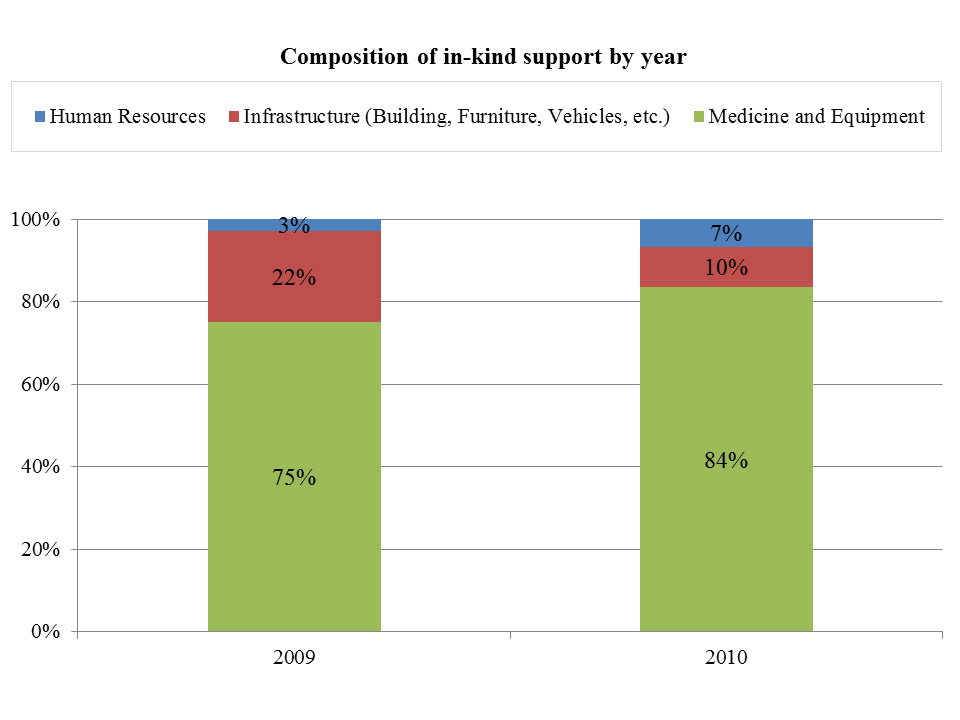

Supplement: Figure S2 — Composition of in-kind support in the 21 health centers. (TIF) [file pmed.1001763.s002.tif]

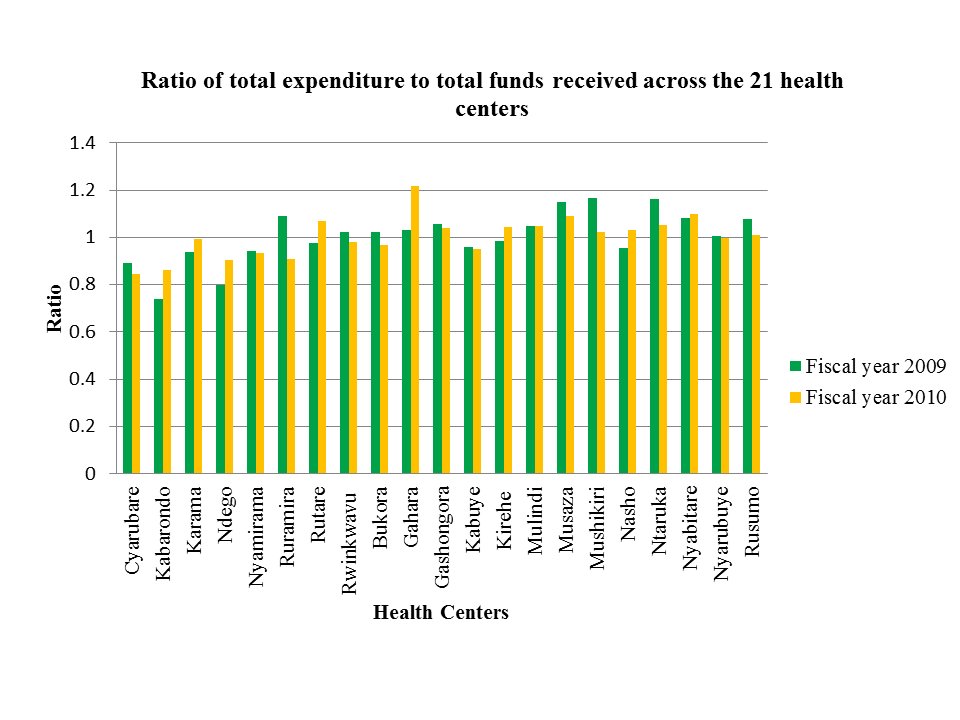

Supplement: Figure S3 — Ratio of total expenditures to total funds received across the 21 health centers. (TIF) [file pmed.1001763.s003.tif]
