# Supplementary material for: Tracking Rural Health Facility Financial Data in Resource-Limited Settings: A Case Study from Rwanda
Source: PLoS Med. 2014 Dec 2;11(12):e1001763. doi: 10.1371/journal.pmed.1001763 (PMC4251825; doi:10.1371/journal.pmed.1001763)
Supplement: Text S1 — Health center financial survey. (DOCX) [file pmed.1001763.s004.docx]

**Supporting Information**

**S1. Health Center Financial Survey**

The Health Center Financial Survey includes three parts: (1) Resource Inputs and Their Sources (Cash), (2) Expenditures (Cash), and (3) In-Kind Supports.

**Part 1 Resource Inputs and Their Sources (Cash)**

This survey is used to collect data on cash funds received by a health center.

Date of Interview (dd/mm/yyyy): [__|__/__|__/__|__|__|__]

Fiscal Year: ___________________________

Name of Interviewer(s): ___________________________

Name of Facility: ___________________________

District: ___________________________

Province: ___________________________

Individuals interviewed:

| Name | Position | Contact information |
| --- | --- | --- |
|  |  |  |
|  |  |  |
|  |  |  |
|  |  |  |
|  |  |  |

Please fill in the tables to the best of your knowledge.

| ***1. Domestic government resource allocation (Cash)*** | |  |
| --- | --- | --- |
| Total cash funds received DIRECTLY from the Rwandan governments.  Please include the Ministry of Health (MOH), Ministry of Finance (MOF), District Office, District Hospital, and any other government sources.  Please also include money from Mutuelles, Rwanda Medical Insurance (RAMA), Military Medical Insurance (M.M.I), Performance Based Financing (PBF), National Malaria Control Program (PNLP), money for Community Health Workers (CHWs), and the Rwanda Development Organization.  ***Please attach a sheet if more space is needed.*** | Source_______________  Source_______________  Source_______________  Source_______________  Source_______________  Source______________ | RWF ____________________    RWF ____________________  RWF_____________________  RWF_____________________  RWF_____________________  RWF_____________________  -9. Don't know (please specify reasons). |
| ***2. Private sources*** |  |  |
| 2.1 Household out-of-pocket health payments, (including the “ticket moderateur,” RAMA and M.M.I. copayments, and payments from uninsured persons). |  | RAMA ___________________  MUTUELLE________________  MMI_____________________  HOUSEHOLD______________  -9. Don't know (please specify reasons). |
| 2.2 Cash funds received directly from domestic NGOS  (Examples: First Lady Foundation, Kabeho Mwana, etc.)  ***Please attach a sheet if more space is needed.*** | NGO1 Name___________  NGO2 Name___________  NGO3 Name___________ | NGO1 RWF__________________  NGO2 RWF _________________  NGO3 RWF__________________  -9. Don't know (please specify reasons). |
| 2.3 Payments from private insurance  (Examples: Rwandan society insurance (SORAS), New Insurance Company of Rwanda (SONARWA), Mediplan (private medical insurance policy), Rwandan company insurance and reinsurance (CORAR)).  ***Please attach a sheet if more space is needed.*** | Source________________  Source _______________  Source_______________ | RWF ____________________  RWF ____________________  RWF ____________________  -9. Don't know (please specify reasons). |
| ***3. External health aid*** |  |  |
| Total cash funds received DIRECTLY from donors (such as Global Fund, Partners in Health, USAID, PIH, etc.)  ***Please attach a sheet if more space is needed.*** | Source_______________  Source_______________  Source_______________  Source_______________  Source_______________  Source_______________ | RWF __________________  RWF __________________  RWF __________________  RWF __________________  RWF __________________  RWF __________________  -9. Don't know (please specify reasons). |
| ***COMMENTS:*** | *Please write down any comments about the data (for example, if certain sections did not have records, or if there was a change in funding sources, or any other factors that may have affected the quality of the data).* | |

**Part 2 Health Center Expenditures (Cash)**

This survey is used to collect data on cash expenditures at a health center. When items were shared with other institutions, we included a line for % usage to capture the expenditures specifically spent by the health center.

Date of Interview (dd/mm/yyyy): [__|__/__|__/__|__|__|__]

Fiscal Year: ___________________________

Name of Interviewer(s): ___________________________

Name of Facility: ___________________________

District: ___________________________

Province: ___________________________

Individuals interviewed:

| Name | Position | Contact information |
| --- | --- | --- |
|  |  |  |
|  |  |  |
|  |  |  |
|  |  |  |
|  |  |  |

Please fill in the tables to the best of your knowledge. ***Please include only expenditures directly paid by your facility.***

| **Cost Categories** | **Examples of cost items** | | |  | |
| --- | --- | --- | --- | --- | --- |
| ***1. Human resources*** | | | | | |
| 1.1 Salary | | Base pay & overtime for your staff to whom you pay directly. | | | TOTAL RWF ________________  -9. Don't know. |
| 1.2 Benefits/allowances | | Housing, meals, etc. | | | TOTAL RWF _________________  -9. Don't know. |
| 1.3 Incentives | | Pay for performance, bonuses, top-up, etc. | | | TOTAL RWF ________________  -9. Don't know. |
| 1.4 Consultant fees | | Advisors, computer programmers, trainers, etc. | | | TOTAL RWF ________________  -9. Don't know. |
| 1.5 Travel costs for training, workshops, conferences | | Per diems, travel allowances, transportation cost, accommodations, etc.  ***Money given to the staff for trainings or workshops only. All other transport should be recorded in 2.2.5.*** | | | TOTAL RWF _______________  -9. Don't know. |
| 1.6 Professional Development | | Scholarships, tuition reimbursement, memberships, conference registration, professional associations, etc. | | | TOTAL RWF _______________  -9. Don't know. |
| 1.7 Fees for Community Health Workers (CHWs) and accompagnateurs (community health promoters) | | All money spent on CHW activities, including trainings, salaries, transport, etc. | | | TOTAL RWF________________  9. Don’t know. |
| 1.8a Other Taxes | | Please include all taxes NOT reported in 1.1-1.7.  Example: Rwanda Social Security Board (RAMA or CSR), Rwanda Revenue Authority (TPR). | | | TOTAL RWF________________  -9. Don't know. |
| 1.8b Other costs related to human resources | | Describe briefly:  Examples: staff parties, picnics | | | TOTAL RWF _______________    -9. Don't know. |
| ***2. Health Service Delivery*** | |  | | |  |
| 2.1.1 Vehicles | | Purchases of new: cars, ambulances, motorcycles, bicycles, etc. | | | TOTAL RWF ______________  % usage__________________    -9. Don't know. |
| 2.1.2 Buildings | | New constructions, renovations, etc. | | |  |
| Name/Function of new buildings  (Examples: dispensary, inpatient unit, etc.) | | Total square meters surface areas (m^2^) | Purchase price | | Useful Life (Years it will remain useful *from the purchase date*). |
| Building 1 | | __________(m^2^)  -9. Don’t know. | RWF _________ -9. Don't know. | | # of years _____  % usage _________________  -9. Don’t know. |
| Building 2 | | __________(m^2^)  -9. Don’t know. | RWF _________ -9. Don't know. | | # of years _____  % usage _________________  -9. Don’t know. |
| Renovation 1 | | __________(m^2^)  -9. Don’t know. | RWF _________ -9. Don't know. | | # of years _____  % usage _________________  -9. Don’t know. |
| Renovation 2 | | __________(m^2^)  -9. Don’t know. | RWF _________ -9. Don't know. | | # of years _____  % usage _________________  -9. Don’t know. |
| ***Please attach a sheet if more space is needed.*** | |  | | |  |
| 2.1.3 Furniture | | Purchases of new: patient beds, chairs, desks, cupboards, sinks, etc. | | | TOTAL RWF_______________  -9. Don't know. |
| 2.1.4 Water, electricity systems | | Description: Water system, electricity system, generators, etc. | Purchase Price | | Useful Life (Years it will remain useful *from purchase date*). |
| Item 1 | |  | RWF ___________    -9. Don't know. | | # of years _____  % usage _________________    -9. Don’t know. |
| Item 2 | |  | RWF ____________  -9. Don't know. | | # of years _____  % usage _________________    -9. Don’t know. |
| Item 3 | |  | RWF ____________  -9. Don't know. | | # of years _____  % usage _________________  -9. Don’t know. |
| ***Please attach a sheet if more space is needed.*** | |  | | |  |
| 2.1.5 Equipment | | Purchases of new: refrigerators, scales, bed nets, large lab equipment such as microscopes, centrifuges, etc. | | | TOTAL RWF________________  -9. Don't know. |
| 2.1.6 Other costs related to capital construction | |  | | | TOTAL RWF _____________  % usage _____________  -9. Don't know. |
| ***2.2. Maintenance and operations (total value of recurring items)*** | | | | | |
| 2.2.1 Maintenance | Costs of maintaining vehicles, buildings, furniture, equipment, etc. | | | | TOTAL RWF _______________  % usage ________________  -9. Don't know. |
| 2.2.2 Insurance | Insurance for vehicles, buildings, furniture, equipment, public liability, etc. | | | | TOTAL RWF ______________  % usage __________________  -9. Don't know. |
| 2.2.3 Rental | Clinic/office space, meeting/training rooms, equipment rental, etc. (Note: put vehicle rentals in 2.2.5). | | | | TOTAL RWF _______________  % usage __________________  -9. Don't know. |
| 2.2.4 Utilities | Electricity, water, gas, solar panels, etc. | | | | TOTAL RWF _______________  % usage __________________  -9. Don't know. |
| 2.2.5 Transportation | Fuel, vehicle rental, other transport costs (motor, bus, etc.)  Money the health center gives to the staff to travel anywhere during work hours. (Example: home visits, money used to transport nurses to give vaccinations, etc.)  Exclude travel costs related to trainings. These should be included in 1.5. | | | | TOTAL RWF________________  % usage ________________  -9. Don't know. |
| 2.2.6 Patient costs | Food, transport reimbursement, incentives (such as payments for taking medicine or returning for re-checks), cash transfers, etc.  . | | | | TOTAL RWF________________  -9. Don't know. |
| 2.2.7 Non-medical supplies | Register books, cleaning supplies, pens, publications, etc.  (Please try to separate printing and copying costs from non-medical supplies). | | | | TOTAL RWF ________________  % usage _________________  -9. Don't know. |
| 2.2.8 Other costs related to maintenance and operation |  | | | | TOTAL RWF_______________  % usage _________________  -9. Don't know. |
| ***3. Medicines, vaccines and technologies*** |  | | | |  |
| 3.1 Drugs | Essential and program medicines, CHW kits, vaccines, etc. | | | | TOTAL RWF________________  -9. Don't know. |
| 3.2 Other medical supplies | Thermometers, blood pressure cuffs, gloves, masks, delivery kits, etc. | | | | TOTAL RWF _______________  -9. Don't know. |
| 3.3 Laboratory supplies | Small supplies such as test kits, slides, etc. (**excluding** the items that have been reported above such as refrigerators, scales, microscopes, etc.) | | | | TOTAL RWF________________  -9. Don't know. |
| 3.4 Other costs that are not listed above but related to medicine products |  | | | | TOTAL RWF _______________  -9. Don't know. |
| ***4. Health Information*** |  | | | |  |
| 4.1 Equipment | Computers, Personal Digital Assistants (PDAs), phones, servers, printers, scanners, satellite dishes, etc. | | | | TOTAL RWF________________  % usage _________________  -9. Don't know. |
| 4.2 Software | Acquisition, maintenance (excluding HR costs listed above), etc. | | | | TOTAL RWF _____________  % usage __________________  -9. Don't know. |
| 4.3 Communications | Monthly telephone bills, cell phone bills, internet air time, etc. | | | | Total RWF________________  % usage ________________  -9. Don't know. |
| 4.4 Printing, copying | Costs to create public education materials, paper, print cartridges, etc. | | | | TOTAL RWF________________  % usage _________________  -9. Don't know. |
| 4.5 Other costs related to health information |  | | | | TOTAL RWF _____________  % usage _______________  -9. Don't know. |
| ***5. Mutuelles*** | | | | | |
| 5.1 Total amount billed to the Mutuelle office in the fiscal year |  | | | | TOTAL RWF _____________  -9. Don't know. |
| 5.2 Total amount received from the Mutuelle Office in the fiscal year |  | | | | TOTAL RWF _______________  -9. Don't know. |
| 5.3 Other costs related to Mutuelles |  | | | | TOTAL RWF ____________  -9. Don't know. |
| ***6. Other costs that are not included in above list*** | Please specify all money spent in here  Activity 1_____________________________  Activity 2_____________________________  Activity 3_____________________________ | | | | RWF1____________________  RWF2____________________  RWF3____________________  TOTAL____________________  -9. Don't know. |
| ***COMMENTS:*** | *Please write down any comments about the data (for example, if certain sections did not have records, or any other factors that may have affected the quality of the data).* | | | | |

**Part 3 In-Kind Supports**

This survey is used to collect data on goods and services donated to a health center.

Date of Interview (dd/mm/yyyy): [__|__/__|__/__|__|__|__]

Fiscal Year: ___________________________

Name of Interviewer(s): ___________________________

Name of Facility: ___________________________

District: ___________________________

Province: ___________________________

Individuals interviewed:

| Name | Position | Contact information |
| --- | --- | --- |
|  |  |  |
|  |  |  |
|  |  |  |
|  |  |  |
|  |  |  |

Please fill in the tables to the best of your knowledge.

| 1. Did you obtain any goods and services DIRECTLY from **the governments or public sectors** in this fiscal year?  Examples: items that you did not paid for using them, such as consultations, staff (including doctors and nurses), services, medicine, vaccines, medical equipment, vehicles, computers, furniture, office supplies, construction, bed nets, contraceptive products, trainings, etc. |  | 1. Yes.  2. No.  -9. Don't know (please specify reasons). |
| --- | --- | --- |
| 2. Please identify ***public institutions*** (such as Ministry of Health, District Office, District Hospital, District Pharmacy, Medical Procurement Division, Vaccine Preventable Disease Division, and any other government sources) that donated goods and services to your facility in this fiscal year, as well as item names, quantities, their market price, and % of usage. |  |  |
| Item name:  Donor name:  -9. Don't know. | Quantity______  % usage______________  -9. Don't know. | Unit price RWF ___________  -9. Don't know. |
| Item name:  Donor name:  -9. Don't know. | Quantity______  % usage______________  -9. Don't know. | Unit price RWF___________  -9. Don't know. |
| Item name:  Donor name:  -9. Don't know. | Quantity______  % usage_______________  -9. Don't know. | Unit price RWF___________  -9. Don't know. |
| ***Please attach a sheet if more space is needed.*** |  |  |
| 3. Did you obtain any goods and services DIRECTLY from **domestic NGOs** this fiscal year?  Examples: items that you did not paid for using them, such as consultations (doctors and nurses), staff, services, medicine, vaccines, medical equipment, vehicles, computers, furniture, office supplies, bed nets, trainings, etc. |  | 1. Yes.  2. No.  -9. Don't know (please specify reasons). |
| 4. Please identify ***domestic NGOs*** that donated goods and services to your facility in this fiscal year, as well as item names, quantities, their market price, and % of usage. |  |  |
| Item name:  Donor name:  -9. Don't know. | Quantity______  % usage_______________  -9. Don't know. | Unit price RWF ___________  -9. Don't know. |
| Item name:  Donor name:  -9. Don't know. | Quantity______  % usage_______________  -9. Don't know. | Unit price RWF___________  -9. Don't know. |
| Item name:  Donor name:  -9. Don't know. | Quantity______  % usage_______________  -9. Don't know. | Unit price RWF___________  -9. Don't know. |
| ***Please attach a sheet if more space is needed.*** |  |  |
| 5. Did you obtain any goods and services DIRECTLY from **foreign donors or NGOs/FBOs funded by foreign donors** directly in this fiscal year?  Examples: items that you did not paid for using them, such as consultations (doctors and nurses), staff, services, medicine, vaccine, medical equipment, vehicles, computers, furniture, construction done by others (buildings, water tanks), generators, renovations, trainings, office supplies, etc. |  | 1. Yes.  2. No.  -9. Don't know. |
| 6. Please identify ***foreign donors (such as Global Fund, USAID, GAVI, etc.) or NGOs/FBOs* supported by foreign donors (such as Partners in Health)** that donated goods and services to your facility in this fiscal year, as well as item names, quantities, their market price, and % of usage. |  |  |
| Item name:  Donor name:  -9. Don't know. | Quantity______  % usage_______________  -9. Don't know. | Unit price RWF___________  -9. Don't know. |
| Item name:  Donor name:  -9. Don't know. | Quantity______  % usage_______________  -9. Don't know. | Unit price RWF___________  -9. Don't know. |
| Item name:  Donor name:  -9. Don't know. | Quantity______  % usage_______________  -9. Don't know. | Unit price RWF___________  -9. Don't know. |
| ***Please attach a sheet if more space is needed.*** |  |  |
| *7. Other sources that are not listed above (Please specify)*  Item name:  Donor name:  -9. Don’t know. | Quantity______  % usage_______________  -9. Don't know. | Unit price RWF__________  -9. Don't know. |
| ***Please attach a sheet if more space is needed.*** |  |  |
| ***COMMENTS:*** | *Please write down any comments about the data (for example, if certain sections did not have records, or if there was a change in staff, or any other factors that may have affected the quality of the data).* | |
