# Supplementary material for: Tracking Rural Health Facility Financial Data in Resource-Limited Settings: A Case Study from Rwanda
Source: PLoS Med. 2014 Dec 2;11(12):e1001763. doi: 10.1371/journal.pmed.1001763 (PMC4251825; doi:10.1371/journal.pmed.1001763)
Supplement: Text S2 — Cost estimation protocols. (DOCX) [file pmed.1001763.s005.docx]

**Supporting Information**

**S2. Cost Estimation Protocols**

The value of in-kind donations is frequently not reported in costing studies as a result of incomplete and irregular documentation. Many donations are not accompanied by invoices or receipts and few accountants are aware of the market price of donated items. To impute the missing values, we develop the following protocols.

When the value of an item is missing at a health facility, we estimate its value based on market price or known costs of identical or similar items documented by other health facilities if the market price is not available. We assume that:

- 1. Items with identical or similar names have identical mean prices, except where noted.
  2. The mean cost of identically-/similarly-named items is constant across geographic areas in a given year.
  3. According to our field reports, market prices in Rwanda do not vary much over blocks of time (2008-2010, 2010-2012, for example), and the variation is mainly due to inflation.

We constructed a database that was compiled of known item costs collected from all health facilities, and sorted them according to item name/function and year of purchase (see Table 1 for an example database).

Table 1 Example Database of Non-Missing Item Costs

| Item  Category | Item Name/  Function | Number of Items | Year  (Purchasing or receiving) | Health Center  Site | Reported Cost (RWF) |
| --- | --- | --- | --- | --- | --- |
| Furniture | Office desk | 1 | 2009 | Mukarange | 26,000 |
| Furniture | Office desk | 1 | 2010 | Nyakabungo | 48,000 |
| Furniture | Office desk | 1 | 2011 | Rusumo | 24,000 |

To estimate the missing cost for identical or similar items, we follow the hierarchical **four-step algorithm** described below:

***Step 1***. When an item with missing cost matched the name and year of items with known costs, the missing cost was estimated as the mean known costs of the items. If there were no items matching the name and year, the algorithm proceeded to the next step.

Formula:

C_miss_T_ = C_mean_T_

Where:

C_miss_T_ = Missing cost of the item in year T

C_mean_T_ =Mean of the known costs of the same/similar items in year T

Table 2 Example 1 for imputation

| Item  Category | Item Name/  Function | Number of Items | Year  of Purchase | Health Center  Site | Reported Cost (RWF) |
| --- | --- | --- | --- | --- | --- |
| Furniture | Office desk | 1 | 2011 | Rusumo | MISSING |
| Furniture | Office desk | 1 | 2011 | Kageyo | 31,000 |
| Furniture | Office desk | 1 | 2011 | Kageyo | 25,000 |

If the cost of a table at Rusumo Health Center was missing in 2011, using the example data from Table 2, the estimated cost in 2011 = (25,000 + 31,000)/2 = 28,000 RWF.

***Step 2***. When an item with missing cost matched the name and year block of items with known costs, the missing cost was estimated as the mean costs of the items in the year block (2008-2010, 2010-2012 for example) after adjusting for currency inflation with the GDP deflators. If there was no item matching the name and year block, the algorithm proceeded to the next step.

Formula:

C_miss_TY_ = C_mean_TY_ *(D_miss_/D_mean_Y_)

Where:

C_miss_TY_ = Missing cost of the item in year block TY
C_mean_TY_ = Mean cost of the same/similar items in year block TY

D_miss_ = GDP deflator of the year with missing cost

D_mean_Y_ = Mean of the GDP deflators in year block TY

Table 3 Example 2 for imputation

| Item  Category | Item Name/  Function | Number of Items | Year  of Purchase | Health Center  Site | Reported Cost (RWF) | GDP Deflator |
| --- | --- | --- | --- | --- | --- | --- |
| Furniture | Office desk | 1 | 2009 | Karama | MISSING | 127.495 |
| Furniture | Office desk | 1 | 2010 | Kageyo | 31,000 | 141.344 |
| Furniture | Office desk | 1 | 2010 | Kageyo | 25,000 | 141.344 |

If the cost of a table at Karama Health Center was missing in 2009 (Table 3), years 2009 and 2010 are in the same year block, the estimated cost in 2009 = [(31000+25000)/2]*(127.495/141.344) = 25,257 RWF.

***Step 3***. When an item with missing cost was purchased in a year between the years of matching items with known costs, we first adjust the inflation for average known costs of the items before *and* after the missing year using GDP deflators. We then used mean costs to interpolate the annual appreciation/depreciation rate. We estimated the cost of the missing item using the known costs of the closest year and the calculated annual appreciation/depreciation rate. If there were no items matching the name from before *and* after the purchase year, the algorithm proceeded to the next step.

Formula:

Part 1. Calculate the average yearly depreciation rate based on the known costs of the items from the closest years before/after the missing year.

R = [(C_mean_adj_A_ - C_mean_adj_B_)/ C_mean_adj_B_]/n

Where:

R = Average yearly depreciation

C_mean_adj_A_ = Adjusted mean cost (with the GDP deflators) from the items in the closest year (year A) after the missing year

C_mean_adj_B_ = Adjusted mean cost (with the GDP deflators) from the items in the closest year (year B) before the missing year

n = number of years between year A and year B

Table 4 Example 3a for imputation

| Item  Category | Item Name/  Function | Number of Items | Year  of Purchase | Health Center  Site | Reported Cost (RWF) | GDP Deflator |
| --- | --- | --- | --- | --- | --- | --- |
| Furniture | Office desk | 1 | 2003 | Rusumo | 24,000 | 74.236 |
| Furniture | Office desk | 1 | 2004 | Ndego | MISSING | 83.963 |
| Furniture | Office desk | 1 | 2009 | Kageyo | 31,000 | 141.344 |
| Furniture | Office desk | 1 | 2009 | Kageyo | 25,000 | 141.344 |

The cost of a table in Ndego was missing in 2004.

The adjusted mean costs in 2003 = 24,000 *(83.963/74.236) = 27,145.

The adjusted mean costs in 2009 = [(31,000 + 25,000]/2]*(83.963/141.344) = 16,633.

Average Yearly Depreciation = [(16,633 – 27,145)/27,145)]/(2009-2003) = -6.45%

Part 2. Calculate the estimated cost based on yearly depreciation rate and closest known mean costs.

C_miss_T_ = C_Y_adj_ *(1 + R)^n^

Where:

C_miss_T_ = Missing cost of the item in year T

C_Y_adj_ = Adjusted mean costs from the closest year (year Y) to missing year T

R = Average yearly depreciation

n = number of years between year T and year Y

Note that the depreciation rate could either be positive or negative, depending on which direction the estimate is being extrapolated.

Example 3b

C_miss_2004_ = C_2003_adj_ *(1 + R)^n^ = 27,145*(1 – 6.45%)^2004-2003^ = 25,394

We use the adjusted mean cost of year 2003 to estimate the missing cost, because it is the closest to the missing year 2004.

***Step 4***. If there were no items matching the name of the item with missing value, the value of the item was listed as missing.
